# Negative Emotional Reactivity and Somatic Symptoms during Adolescence Predict Adult Health and Wellbeing in Early and Middle Adulthood

**DOI:** 10.1007/s10964-024-01940-9

**Published:** 2024-01-28

**Authors:** Mathias Allemand, Helmut A. Fend, Patrick L. Hill

**Affiliations:** 1 University Research Priority Program “Dynamics of Healthy Aging”, University of Zurich, Zurich, Switzerland; 2 Institute of Education, University of Zurich, Zurich, Switzerland; 3 Department of Psychological and Brain Sciences, Washington University in St. Louis, St. Louis, USA

**Keywords:** Negative emotional reactivity, Somatic symptoms, Adolescent within-person developmental processes, Adult health and wellbeing, Latent Curve Model with Structured Residuals (LCM-SR)

## Abstract

Longitudinal research is lacking with respect to how negative emotional reactivity and somatic symptoms during adolescence set the stage for later health. The aim of this longitudinal study was to examine within-person associations between negative emotional reactivity and somatic symptoms during adolescence and their effects on health and wellbeing in adulthood. Participants (*N* = 1527; 48.3% female) were assessed annually at the age of 12 to 16 years and at the age of 35 and 45 years. Adolescents with frequent somatic symptoms reported higher reactivity. Individual differences in levels and changes of somatic symptoms and reactivity were independently associated with adult health and wellbeing decades later. The findings underscore the importance of considering how individual differences change during adolescent development.

## Introduction

Adults who are more emotionally reactive tend to experience worse physical health outcomes, as evident in the work linking higher neuroticism (a dispositional tendency toward negative emotions and reactivity) to greater reports of somatic symptoms (Costa & McCrae, 1985; Leger et al., 2021). Most of this literature though focuses on adult samples, and there are reasons to believe that negative emotional reactivity may have similar, or more muted effects earlier in the lifespan. For instance, adolescence is a period of widespread development of affective processing systems (Casey et al., 2008; Silvers, 2022), and as such, negative emotional reactivity may be more common and less maladaptive as a result. Even if negative emotional reactivity retained health consequences in adolescence, it may fail to demonstrate downstream consequences on later adult outcomes, given the perceived “normative” nature of reactivity in adolescence. The current study investigated bidirectional associations between somatic symptoms, or reports of physical pain and other health symptoms, and negative emotional reactivity, defined as a tendency to respond with negative emotions to stressors, unpleasant events and challenges, during adolescence (ages 12 to 16 years). In addition, this study examined how adolescent levels for negative emotional reactivity and somatic symptoms influenced later wellbeing and health outcomes in adulthood (ages 35 and 45). In so doing, the current study advances our understanding of how adolescent dispositional tendencies may hold concurrent and later influence on health and wellbeing, providing an empirical foundation for potential intervention efforts.

### Negative Emotional Reactivity and Somatic Symptoms

Decades of health psychology research have pointed to the value of considering whether people perceive themselves as more or less emotionally reactive. Most contemporary research has centered on the health consequences of neuroticism, a personality trait defined by greater depressive and anxiety symptoms, emotional lability, and volatility (Friedman, 2019; John et al., 2008). As such, this trait can be seen as including negative emotional reactivity, in addition to a focus on greater negative emotionality in general. Greater neuroticism has been linked to worse physical health outcomes, including earlier mortality risk (Mroczek & Spiro, 2007; Shipley et al., 2007). Critical to the study of somatic symptoms, adults higher on neuroticism also tend to report greater physical issues. Studies have linked neuroticism to worse self-rated health and greater physical symptom counts (Rosmalen et al., 2007; Stephan et al., 2020). Theoretical rationale behind these findings points to at least two arguments: first, more reactive adults are more vigilant and attuned to potential issues compared to others, and second, negative emotional reactivity can disrupt social relationships, leading adults to view less support and in turn control of issues when they do arise. As such, adults higher on negative emotional reactivity may be more likely to report symptoms and experience them.

Although this research has focused on adult samples, a handful of adolescent studies support similar claims. For instance, neuroticism predicts pain reports among adolescents and reports of somatic symptoms (Murberg & Bru, 2007; Wilner et al., 2014). The relative paucity of work compared to adulthood though may result more from terminological differences, as adolescent researchers often avoid Big Five personality traits to focus on constructs such as negative emotional reactivity or emotion regulation, which may be seen as precursors to these trait domains later in adulthood. Studies on these fronts also find reports of poorer wellbeing and greater health issues among those adolescents who report being more emotionally reactive (Myerberg et al., 2019), leading to efforts to implement emotional regulation interventions to promote adolescent health (Horn et al., 2011; Houck et al., 2016). However, few studies have examined these associations over time, allowing for the potential to investigate directionality of the claims. Moreover, the associations may vary as a function of gender. Research has shown that somatic symptoms are particularly prevalent in adolescent girls (Steinhausen & Winkler Metzke, 2007; Swain et al., 2014). There is additional evidence that girls become increasingly more prone to negative affect, a facet of neuroticism (Soto, 2016; Van den Akker et al., 2014).

### Considering Directionality between Negative Emotional Reactivity and Somatic Symptoms

Researchers have typically tested or simply assumed that the directionality of these associations went from reactivity (or neuroticism) to the health outcomes of interest. That said, being a more emotionally reactive individual can manifest in somatic issues. The focus on negative emotional reactivity as the predictor comes from what may appear as the most logical direction. However, researchers have critiqued the literature for not considering the potential for health issues to influence changes in dispositions (Mroczek et al., 2020). That is, frequent somatic symptoms can lead to increased negative emotional reactivity. Adolescent literature has accumulated showing how depressive symptoms, anxiety, and hostility can yield objective physical health consequences later (Ames & Leadbeater, 2018; Räikkönen et al., 2003), dating back to the initial evidence for mind-body connections. However, there are reasons why somatic symptoms may change one’s level of reactivity over time. If concerns are linked to objective health issues, then it can cause accumulating distress for the individual, leading them to be more emotionally reactive. This response, in fact, can be seen as adaptive, insofar that they may need to be more vigilant and reactive in the future, if they are at risk for developing health concerns. This logic has been the root of the research into whether aspects of neuroticism could in fact be healthy (Friedman, 2019), which has yielded, at best, mixed results (Graham et al., 2020; Weston et al. (2020)). During adolescence, though, there are additional reasons to believe somatic symptoms could yield dispositional changes. Adolescence is a period of widespread identity development (Erikson, 1959; Marcia, 1980). Health issues can present a challenge to this belief, and perhaps lead the adolescent to lower sense of self when compared to their relatively healthy peers. As such, somatic symptoms in adolescence could yield increases in negative emotional reactivity. The current study thus provides a substantive advance to the field by examining directionality in both directions using five waves of data during adolescence. Moreover, it was possible to consider whether fluctuations in negative emotional reactivity and somatic symptoms during adolescence held consequences in the longer term for adult health and wellbeing.

### Long-Term Associations with Adult Health and Wellbeing

A lifespan developmental perspective underscores how our early environments, psychosocial characteristics, and challenges chart the course for our future. Within health psychology, researchers have supported this claim in multiple ways. For instance, childhood health behaviors are related to propensity to enact similar behaviors later in life. Being active early in the lifespan predicts greater physical activity in adulthood (Malina, 2001; Telama et al., 2005). Eating behaviors in childhood predict risk for disordered eating in adolescence (Herle et al., 2020). In turn, adolescent disordered eating predicts greater likelihood for eating disorders in adulthood (Kotler et al., 2001).

Researchers also have described how levels and changes in dispositional characteristics during childhood and adolescence may set the stage for later health concerns. Theoretical work has suggested at least three pathways by which these downstream consequences may occur (Hill et al., 2019). First, dispositional traits influence the health behavior decisions one makes (Bogg & Roberts, 2004; Smith, 2006). Being more emotionally reactive can lead adolescents to take riskier health decisions (e.g., substance use, criminal activity, etc.) that lead to consequences that can result in long-term health problems. Second, given the capacity for individual differences in personality change during adolescence (Allemand et al., 2019; Brandes et al., 2021), it is important to consider which adolescents develop in a more or less adaptive manner. Moreover, there likely are lasting benefits to developing emotional stability earlier than one’s peers. In the adult literature, evidence on this front comes from work showing that changes in neuroticism are predictive of later health behaviors and outcomes, even when accounting for one’s initial level of neuroticism (Mroczek & Spiro, 2007; Turiano et al., 2012). Third, dispositional traits may hold age-specific consequences for health outcomes (Shanahan et al., 2014). Being emotionally reactive can influence different health behaviors across the lifespan, depending on the developmental context; for example, it may impact age of first drug use early in life versus propensity to seek healthcare later in adulthood.

Multiple studies have now demonstrated how changes during adolescence predict later adult outcomes, including social functioning (Allemand et al., 2015) and depression (Allemand et al., 2022; Steiger et al., 2014). Past work with the current study sample, for instance, has focused on self-control tendencies during adolescence. Adolescents higher on self-control, as well as those who *increased* on the disposition over time, were shown to be more forgiving later in middle adulthood (Allemand et al., 2023). Similar level and change associations also were shown when predicting relationship and occupational levels later in life (Allemand et al., 2019), again with benefits for adolescents who started with and developed greater self-control. However, work with this sample has yet to consider the role of negative emotional reactivity on later outcomes, including concurrent or future somatic symptoms.

## Current Study

The current study sought to address the relative paucity of work on negative emotional reactivity and somatic symptoms during adolescence, by examining their bidirectional associations and whether they predict somatic symptoms later in adulthood. This study employed data from a longitudinal study of German adolescents that started in 1979; although every study must be considered within its sociohistorical context, it is unclear whether the current context would influence the associations tested herein. The current study had four aims. The first aim was to examine within-person bidirectional associations between somatic symptoms and negative emotional reactivity across adolescence from age 12 to age 16. It was hypothesized that higher somatic symptoms would be associated with higher negative emotional reactivity in adolescence. In addition, it was hypothesized that somatic symptoms and negative emotional reactivity would concurrently show positive within-person associations at each measurement occasion. The second aim was to examine prospective associations of individual differences in level and change in somatic symptoms and negative emotional reactivity across adolescence with somatic symptoms at the age of 45. It was hypothesized that higher levels as well as increases during adolescence would be associated with more somatic symptoms in middle adulthood. Because negative emotional reactivity was not measured at age 45, it was not possible to examine prospective associations with negative emotional reactivity in middle adulthood. The third aim was to examine the prospective associations of level and change in somatic symptoms and negative emotional reactivity in adolescence with somatic symptoms in middle adulthood when accounted for satisfaction with life and health, and health impairment at the age of 35. Satisfaction with life and health as well as health impairment were used as secondary health and wellbeing outcome variables, as both negative emotional reactivity and somatic complaints were not measured at the age of 35. The fourth aim was to test the robustness of the results by including gender as a time-invariant covariate.

## Methods

### Participants and Procedure

Data from the German LifE-Study (Lebensverläufe von der späten Kindheit ins frühe Erwachsenenalter [Pathways From Late Childhood to Adulthood]; Fend et al., 2002) were used, in which adolescents were assessed five times during adolescence: at the age of 12 years (1979; *N* = 2054), 13 years (1980; *N* = 2047), 14 years (1981; *N* = 2003), 15 years (1982; *N* = 1952), and 16 years (1983; *N* = 1790). Two follow-up assessments were conducted in adulthood. The first follow-up assessment was conducted when participants were 35 years old (2002; *N* = 1527). Of these participants, 48.3% were female, 59.2% were married, 32.8% were single, 7.8% were either divorced or separated, and 0.1% of the participants were widowed. The second follow-up assessment was conducted when participants were 45 years old (2012; *N* = 1359). Of these participants, 50.6% were female, 65.3% were married, 18.6% were single, 15.5% were divorced or separated, and 0.7% of the participants were widowed. Because a main part of this study is the prediction of health and wellbeing in adulthood, the focus was on those participants who participated at least at the age of 35 in adulthood. For more information on the LifE-Study and participants, sampling procedure, and attrition, see Fend et al. (2009) and Steiger et al. (2015).^1^

### Predictor Measures in Adolescence

#### Somatic symptoms

From age 12 to 16, participants were asked how often they had the following five somatic symptoms: (a) headache, (b) stomach or abdominal pain, (c) indigestion (e.g., nausea and vomiting), (d) difficulty falling asleep or sleeping through the night, (e) circulatory problems (e.g., dizziness or feeling “black from the eyes”). Participants rated each symptom on a 5-point scale (1 = never, 2 = a few times a year, 3 = several times a month, 4 = several times a week, 5 = even more often). A high score indicates frequent somatic symptoms. The alpha reliability estimates ranged from 0.66 to 0.74 for the five measurement occasions.

#### Negative emotional reactivity

From age 12 to 16, participants answered 8 items measuring negative emotional reactivity (Fend, 1994; Fend & Prester, 1986). Sample items are “Sometimes I get upset about every little thing”; “Sometimes I don’t know what’s going on with me”; “Sometimes I feel very sad for no important reason”. All items are shown in Appendix 1. Participants rated each item on a dichotomous response scale (1 = not true, 2 = true). High scores indicate higher negative emotional reactivity. The reliability estimates (Kuder–Richardson KR-20) ranged from 0.75 to 0.79 for the five measurement occasions.

### Outcome Measures in Adulthood at Age 45

While somatic symptoms were assessed at age 45, negative emotional reactivity was not assessed. The same five items were used to measure *somatic symptoms* as in adolescence, plus two additional symptoms: (a) nervousness and (b) difficulty concentrating. Participants rated each symptom on a 5-point scale (1 = never, 2 = a few times a year, 3 = several times a month, 4 = several times a week, 5 = even more often). The alpha reliability estimates for the 7-item scale was 0.78. The alpha reliability estimates for the scale without the two additional items was 0.68. This scale and the scale without the two additional symptoms were very highly correlated (*r* = 0.94, *p* < 0.001).

### Outcome Measures in Adulthood at Age 35

As somatic symptoms and negative emotional reactivity were not assessed at the age of 35, three indicators of health and well-being were used as outcome variables: (1) *Satisfaction with life* was measured with a single item in which participants were asked about their overall satisfaction with their lives on a 7-point scale (1 = extremely dissatisfied to 7 = extremely satisfied). (2) *Satisfaction with health* was measured by asking participants how good they felt about their overall health on a 5-point scale (1 = not good at all to 5 = very good). (3) *Health impairment* was measured with a single item in which participants were asked on a dichotomous scale (1 = not impaired, 2 = impaired) whether they felt their health was impaired.

### Statistical Analysis

Longitudinal structural equation modeling (SEM) was used to examine how negative emotional reactivity and somatic symptoms jointly unfold from age 12 to age 16 and how these relations vary within and across adolescents. Specifically, the Latent Curve Model with Structured Residuals (LCM-SR; Curran et al., 2014) was used. This model provides simultaneous estimates of person-specific, between-person change processes and time-specific, within-person change processes of the relation between negative emotional reactivity and somatic symptoms over time.

The LCM-SR captures between-person differences in change by incorporating latent intercepts and latent slope factors for negative emotional reactivity and somatic symptoms, similar to classic latent change models. In addition, LCM-SR estimates three types of within-person differences in change: autoregressive, cross-lagged, and concurrent processes. The *autoregressive* parameters represent the within-person stability associations of the constructs. For example, a significant and substantial autoregressive effect of somatic symptoms would indicate strong stability between assessments. The *cross-lagged* regression parameters evaluate the extent to which within-person change in somatic symptoms is associated with the individual’s prior negative emotional reactivity, and the extent to which within-person change in negative emotional reactivity is associated at the previous measurement occasion. For example, a positive cross-lagged effect between somatic symptoms and negative emotional reactivity would suggest that somatic symptoms above the individual’s own average at one time point is associated with a subsequent above-the-average score of negative emotional reactivity at the next time point. The *concurrent* parameters refer to correlated change (i.e., change correlations between residuals) between the time-specific residuals of somatic symptoms and negative emotional reactivity when their autoregressive effects and cross-lagged associations are controlled. For example, a positive concurrent association between somatic symptoms and negative emotional reactivity would show to what extent deviations of somatic symptoms from the person-specific average are accompanied by deviations of negative emotional reactivity from the person-specific average.

**Fig. 1:**
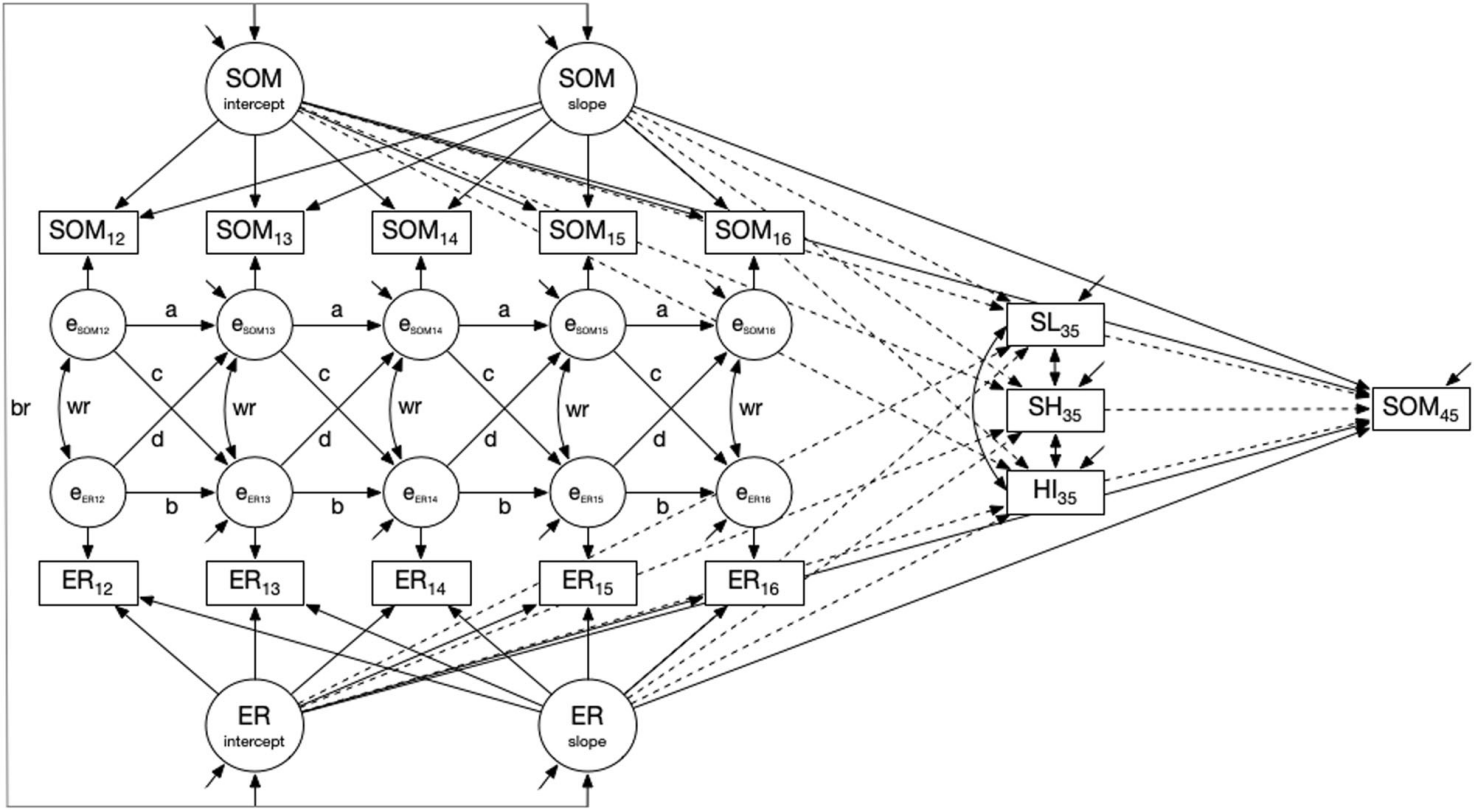
Bivariate unconditional LCM-SR for somatic symptoms and negative emotional reactivity in adolescence and adult health and wellbeing (Model M9). The conceptual model includes the manifest indicators of somatic symptoms (SOM_12_ to SOM_16_) and negative emotional reactivity (ER_12_ to ER_16_) from age 12 to 16, the related time-specific residuals (*e*_SOM12_ to *e*_SOM16_ and *e*_ER12_ to *e*_ER16_), the within-person autoregressive effects for somatic symptoms and negative emotional reactivity (**a**, **b**), the within-person cross-lagged effects (**c**, **d**), the within-person concurrent relations and the concurrent correlation at age 12 (wr), satisfaction with life at age 35 (SL_35_), satisfaction with health (SH_35_), and health impairment (HI_35_), and somatic symptoms at age 45 (SOM_45_). The autoregressive and cross-lagged effects and the concurrent relations are constrained to be equal over time with the exception of the concurrent correlation at age 12. The model also tested the between-person associations (br) between the intercept and slope factors of somatic symptoms (SOM_intercept_ and SOM_slope_) and negative emotional reactivity (ER_intercept_ and ER_slope_). To make the figure clearer, the predictive effects of the intercept and slope factors of somatic symptoms and negative emotional reactivity on the outcome variables at age 35 and the predictive effects of the variables at age 35 on somatic symptoms at age 45 are plotted as dashed lines. The conceptual model without the dashed lines and without the variables at age 35 reflects Model M8.

The analyses largely followed the model-building-strategy proposed by Curran et al. (2014). The first step was to establish an *univariate unconditional LCM-SR* separately for somatic symptoms and negative emotional reactivity. This included estimating an intercept-only model (M1, M4) and a linear change model (M2, M5) and testing of autoregressions among the residuals (M3, M6). Given that there were no theoretical reasons to expect differential autoregressive effects from ages 12 to 16, these parameters were constrained to be equal over time. The second step was to estimate a *bivariate unconditional LCM-SR* that combines both univariate models (M7). Given that there were no theoretical reasons to expect differential cross-lagged effects from ages 12 to 16, these parameters were constrained to be equal over time. Similarly, the concurrent associations were constrained to be equal from age 13 to 16 (see also Curran et al., 2014). The third step included the regression of somatic symptoms in middle adulthood on the latent intercept and slope factors of somatic symptoms and negative emotional reactivity in adolescence (M8). Next, satisfaction with life and health, and health impairment at the age of 35 were additionally included as predictors of somatic symptoms at the age of 45 in addition to the intercept and slope factors of somatic symptoms and negative emotional reactivity as predictor variables from adolescence (M9). This model is illustrated in Fig. 1. In this model, satisfaction with life and health and health impairment were regressed on the intercept and slope factors of somatic symptoms and negative emotional reactivity. The fourth step included two additional *bivariate conditional LCM-SR* models based on models M8 and M9 with gender (1 = female, 2 = male) as a time-invariant covariate to account for possible gender effects (M10, M11). These two models served as sensitivity analyses.

Analyses were performed with Mplus 8 (Muthén & Muthén, 1998–2017) using full-information likelihood (FIML). To evaluate goodness of fit of the models, the chi-square (χ^2^), comparative fit index (CFI), and root mean square error of approximation (RMSEA) were examined. CFI values above 0.97 and RMSEA values below 0.06 are considered to reflect a good model fit (Hu & Bentler, 1999). Moreover, 95% confidence intervals (CIs) for the unstandardized estimates, standard errors, and *p*-values from the LCM-SRs as well as the standardized estimates were reported.

## Results

Descriptive statistics and zero-order correlations of the main variables are shown in Table 1. The year-to-year stability correlations for somatic symptoms ranged from 0.51 to 0.68 and for negative emotional reactivity ranged from 0.52 to 0.63 (all *p* < 0.001). The results indicated positive associations between somatic symptoms and negative emotional reactivity across adulthood. Moreover, the results suggested that more somatic symptoms during adolescence were associated with more somatic symptoms at the age of 45 years with associations in the range of 0.19 to 0.42 (all *p* < 0.001). Higher negative emotional reactivity in adolescence was associated with more somatic symptoms in middle adulthood with correlations ranging between 0.16 to 0.26 (all *p* < 0.001).

**Table 1:** Descriptive statistics and zero-order correlations among the predictor and outcome variables

Variables	1	2	3	4	5	6	7	8	9	10	11	12	13	14
1. Somatic symptoms age 12	–													
2. Somatic symptoms age 13	0.51*	–												
3. Somatic symptoms age 14	0.48*	0.62*	–											
4. Somatic symptoms age 15	0.38*	0.51*	0.59*	–										
5. Somatic symptoms age 16	0.45*	0.55*	0.64*	0.68*	–									
6. Negative emotional reactivity age 12	0.31*	0.23*	0.21*	0.21*	0.25*	–								
7. Negative emotional reactivity age 13	0.24*	0.30*	0.26*	0.27*	0.26*	0.52*	–							
8. Negative emotional reactivity age 14	0.22*	0.30*	0.33*	0.26*	0.32*	0.46*	0.57*	–						
9. Negative emotional reactivity age 15	0.19*	0.24*	0.29*	0.31*	0.30*	0.36*	0.50*	0.60*	–					
10. Negative emotional reactivity age 16	0.22*	0.25*	0.33*	0.31*	0.38*	0.39*	0.48*	0.58*	0.63*	–				
11. Satisfaction with life age 35	−0.12*	−0.11*	−0.11*	−0.15*	−0.12*	−0.15*	−0.13*	−0.16*	−0.14*	−0.23*	–			
12. Satisfaction with health age 35	−0.12*	−0.16*	−0.15*	−0.15*	−0.21*	−0.12*	−0.14*	−0.15*	−0.12*	−0.15*	0.47*	–		
13. Health impairment age 35	0.09*	0.10*	0.10*	0.11*	0.14*	0.08*	0.08*	0.11*	0.09*	0.11*	−0.33*	−0.49*	–	
14. Somatic symptoms age 45	0.19*	0.26*	0.27*	0.28*	0.42*	0.16*	0.17*	0.19*	0.23*	0.26*	−0.30*	−0.35*	0.30*	–
Potential range	1–5	1–5	1–5	1–5	1–5	1–2	1–2	1–2	1–2	1–2	1–7	1–5	1–2	1–5
*M*	1.96	1.99	2.03	2.09	2.08	1.49	1.51	1.51	1.50	1.48	5.57	3.89	1.32	2.03
*SD*	0.61	0.58	0.60	0.64	0.56	0.29	0.30	0.30	0.31	0.31	1.17	0.72	0.47	0.58

*p < 0.001

### Univariate Unconditional LCM-SR for Somatic Symptoms

The analyses started with a random intercept model (M1) for somatic symptoms that included only a mean and variance of the intercept factor and residual variances for each of the repeated measures that were allowed to vary over time. This model fitted the data poorly (Table 2). This model was expanded with the addition of a linear slope factor (M2) by fixing the slope factor loadings to 0, 1, 2, 3, and 4 to represent the annually assessments. A mean and a variance were estimated for the intercept and slope factors, and time-specific residual variances were allowed to vary over time. This model reflected a good fit to the data and resulted in a significant improvement in model fit relative to the intercept-only model. The model fit is shown in Table 2. The mean and variance were significant for both the intercept (*M* = 1.97, *SE* = 0.02, *p* < 0.001; *Var* = 0.18, *SE* = 0.01, *p* < 0.001) and linear slope (*M* = 0.03, *SE* = 0.01, *p* < 0.001, *Var* = 0.01, *SE* = 0.001, *p* < 0.001), respectively. These results indicate that participants’ levels of somatic symptoms significantly increased at a linear rate of change, and that there was significant variability around both the intercept and rate of change over time. Finally, this model was expanded by adding an autoregressive component among the residuals (M3). The autoregressive parameter was significant (*B* = 0.09, *SE* = 0.04, *p* = 0.02, *β*s = 0.08 to 0.14), and the model fit was significantly improved with the inclusion of the autoregressive residual structure (Table 2).

**Table 2:** Model fit information for the latent curve models with structured residuals (LCM-SR)

Model	χ^2^	df	CFI	RMSEA (90% CI)	Δχ^2^	Δdf
*Univariate unconditional LCM-SR for somatic symptoms*						
M1: Intercept only model	184.11***	13	0.911	0.093 (0.081; 0.105)	–	–
M2: Linear change model	37.39***	10	0.986	0.042 (0.028; 0.057)	146.72***	3
M3: Linear change model plus equal autoregressive effects	31.87***	9	0.988	0.041 (0.026; 0.057)	5.52*	1
*Univariate unconditional LCM-SR for negative emotional reactivity*						
M4: Intercept only model	139.76***	13	0.930	0.080 (0.068; 0.092)	–	–
M5: Linear change model	44.00***	10	0.981	0.047 (0.033; 0.062)	95.76***	3
M6: Linear change model plus equal autoregressive effects	27.41**	9	0.990	0.037 (0.021; 0.053)	16.59***	1
*Bivariate unconditional LCM-SR for somatic symptoms and negative emotional reactivity*						
M7: Combination of M3 and M6 plus equal cross-lagged regressions and concurrent residual associations^a^	66.28**	35	0.992	0.024 (0.015; 0.033)	–	–
M8: M7 plus somatic symptoms age 45	72.73**	41	0.993	0.023 (0.014; 0.031)	–	–
M9: M8 plus satisfaction with life and health, health impairment age 35 and somatic symptoms age 45	95.31**	59	0.993	0.020 (0.012; 0.027)	–	–
*Bivariate conditional LCM-SR for somatic symptoms and negative emotional reactivity*						
M10: M8 plus gender	75.81**	47	0.994	0.020 (0.011; 0.028)	–	–
M11: M9 plus gender	97.81**	65	0.994	0.018 (0.010; 0.025)	–	–

*p < 0.05; **p < 0.01; ***p < 0.001

^a^The concurrent residual association at the age 12 was freely estimated

### Univariate Unconditional LCM-SR for Negative Emotional Reactivity

The results of the intercept-only model (M4) and linear change model (M5) are shown in Table 2. Again, the linear change model reflected a good fit to the data and resulted in a significant improvement in model fit relative to the intercept-only model (Table 2). Both the mean and variance were significant for the intercept (*M* = 1.50, *SE* = 0.01, *p* < 0.001; *Var* = 0.05, *SE* = 0.003, *p* < 0.001). The mean for the linear slope factor was not significant, but the variance was (*M* = 0.003, *SE* = 0.003, *p* = 0.23; *Var* = 0.003, *SE* < 0.001, *p* < 0.001). These results indicated that, on average, negative emotional reactivity was stable during adolescence, but participants differed in their intraindividual rates of change. As such, the linear slope factor was retained. The inclusion of time-adjacent autoregressions among residuals (M6) led to a significant improvement in model fit (Table 2), with a significant autoregressive parameter (*B* = 0.16, *SE* = 0.04, *p* < 0.001, *β*s = 0.15 to 0.17).

### Bivariate Unconditional LCM-SR for Somatic Symptoms and Negative Emotional Reactivity

Two univariate LCM-SRs with the autoregressive components between the residuals of somatic symptoms and negative emotional reactivity (i.e., autoregressive parameters) were combined in a single bivariate model (M7). The intercept and slope factors of somatic symptoms were allowed to covary with the intercept and slope factors of negative emotional reactivity. Equal cross-lagged regression parameters over time were estimated and the time-specific residuals were allowed to covary between somatic symptoms and negative emotional reactivity. These covariances were set to be equal across age 13 to 16 (i.e., concurrent parameters) and freely estimated the first residual correlation at the age of 12. The model fit the data well (Table 2). The parameters from this model M7 can be shown in Table 3. The significant autoregressive parameters showed low within-person stability of somatic symptoms and negative emotional reactivity, controlling for the stable levels (i.e., between-person differences in levels and slopes). The results of the concurrent associations have shown that at the individual level somatic symptoms covary with negative emotional reactivity. The two constructs showed correlated changes, but were not related in terms of lagged effects. A strong correlation between the random intercepts of somatic symptoms and negative emotional reactivity was found at the between-person level, indicating that those participants with higher scores in somatic symptoms during adolescence tended to also report higher negative emotional reactivity (Table 3).

**Table 3:** Parameter estimates from the bivariate unconditional LCM-SR for somatic symptoms and negative emotional reactivity in adolescence (Model M7)

Parameter	Estimate	95% CI LL	95% CI UL	*SE*	*p*	Std. Est.
*Within-person autoregressive relations*						
Somatic symptoms	0.09	0.01	0.17	0.04	0.02	0.08 to 0.14
Negative emotional reactivity	0.15	0.07	0.23	0.04	0.000	0.14 to 0.16
*Within-person cross-lagged effects*						
Somatic symptoms → negative emotional reactivity	0.02	−0.01	0.05	0.02	0.22	0.03 to 0.04
Negative emotional reactivity → somatic symptoms	−0.004	−0.12	0.11	0.06	0.94	−0.002 to −0.003
*Within-person concurrent relations*						
Somatic symptoms ↔ negative emotional reactivity age 12	0.02	0.01	0.03	0.01	0.000	0.23
Somatic symptoms ↔ negative emotional reactivity age 13 to 16	0.01	0.003	0.02	0.002	0.001	0.09 to 0.16
*Between-person relations between intercepts and slope factors*						
Intercept of SC ↔ slope of SC	−0.002	−0.01	0.01	0.01	0.68	−0.06
Intercept of ER ↔ slope of ER	−0.002	−0.01	0.001	0.001	0.12	−0.21
Intercept of SC ↔ intercept of ER	0.04	0.03	0.05	0.01	0.000	0.48
Intercept of SC ↔ slope of ER	0.002	−0.002	0.005	0.002	0.37	0.10
Slope of SC ↔ intercept of ER	0.000	−0.003	0.004	0.002	0.83	0.02
Slope of SC ↔ slope of ER	0.001	0.000	0.002	0.001	0.16	0.24

→ denotes a regression path; ↔ denotes a covariation/correlation

### Long-Term Associations with Somatic Symptoms at Age 45

Next, the outcome variable somatic symptoms in middle adulthood was regressed on the latent intercept and slope factors of somatic symptoms and negative emotional reactivity in adolescence (M8). This model fit the data well (Table 2). Results suggested that individual differences in level and change of somatic symptoms were positively related to somatic symptoms in middle adulthood (Table 4). These results demonstrated that change (slope) in somatic symptoms and level (intercept) were independently related to somatic symptoms at age 45, indicating that those participants who reported more somatic symptoms in adolescence, and who showed an increase across the adolescent years, reported the highest levels of somatic symptoms in middle adulthood. The level of negative emotional reactivity was also positively related to somatic symptoms in middle adulthood (Table 4). In addition, the slope of negative emotional reactivity was significantly associated with adult somatic symptoms (Table 4), indicating that increases in negative emotional reactivity were associated with higher reported somatic symptoms in adulthood. Note that somatic symptoms at age 45 were simultaneously regressed on the intercept and slope factors of somatic symptoms and negative emotional reactivity.

**Table 4:** Selected parameter estimates from the bivariate unconditional LCM-SR for somatic symptoms and negative emotional reactivity in adolescence and adult health and wellbeing (Models M8 and M9)

Parameter	Estimate	95% CI LL	95% CI UL	*SE*	*p*	Std. Est.
*Model M8*						
Intercept of somatic symptoms → somatic symptoms age 45	0.34	0.20	0.48	0.07	0.000	0.23
Intercept of negative emotional reactivity → somatic symptoms age 45	0.40	0.17	0.63	0.12	0.001	0.14
Slope of somatic symptoms → somatic symptoms age 45	1.93	0.98	2.87	0.48	0.000	0.28
Slope of negative emotional reactivity → somatic symptoms age 45	1.90	0.12	3.68	0.91	0.04	0.15
*Model M9*						
Intercept of somatic symptoms → satisfaction with life age 35	−0.16	−0.42	0.10	0.13	0.23	−0.05
Intercept of somatic symptoms → satisfaction with health age 35	−0.24	−0.40	−0.08	0.08	0.003	−0.13
Intercept of somatic symptoms → health impairment age 35	0.12	0.02	0.23	0.05	0.02	0.10
**Intercept of somatic symptoms → somatic symptoms age 45**	0.29	0.16	0.42	0.07	0.000	0.19
Slope of somatic symptoms → satisfaction with life age 35	−0.15	−1.60	1.31	0.74	0.84	−0.01
Slope of somatic symptoms → satisfaction with health age 35	−1.02	−1.93	−0.12	0.46	0.03	−0.12
Slope of somatic symptoms → health impairment age 35	0.19	−0.37	0.75	0.29	0.50	0.04
**Slope of somatic symptoms → somatic symptoms age 45**	1.73	0.85	2.61	0.45	0.000	0.25
Intercept of negative emotional reactivity → satisfaction with life age 35	−1.07	−1.52	−0.63	0.23	0.000	−0.19
Intercept of negative emotional reactivity → satisfaction with health age 35	−0.37	−0.64	−0.10	0.14	0.008	−0.11
Intercept of negative emotional reactivity → health impairment age 35	0.17	−0.01	0.35	0.09	0.06	0.08
**Intercept of negative emotional reactivity → somatic symptoms age 45**	0.27	0.05	0.49	0.11	0.02	0.10
Slope of negative emotional reactivity → satisfaction with life age 35	−3.81	−6.96	−0.67	1.60	0.02	−0.15
Slope of negative emotional reactivity → satisfaction with health age 35	−0.89	−2.75	0.97	0.95	0.35	−0.06
Slope of negative emotional reactivity → health impairment age 35	0.19	−1.00	1.38	0.61	0.75	0.02
**Slope of negative emotional reactivity → somatic symptoms age 45**	1.54	−0.12	3.21	0.85	0.07	0.12
Satisfaction with life age 35 → somatic symptoms age 45	−0.06	−0.09	−0.03	0.02	0.000	−0.12
Satisfaction with health age 35 → somatic symptoms age 45	−0.12	−0.17	−0.07	0.03	0.000	−0.15
Health impairment age 35 → somatic symptoms age 45	0.19	0.12	0.26	0.04	0.000	0.15
Satisfaction with life age 35 ↔ satisfaction with health age 35	0.35	0.31	0.40	0.02	0.000	0.45
Satisfaction with life age 35 ↔ health impairment age 35	−0.15	−0.17	−0.13	0.01	0.000	−0.31
Satisfaction with health age 35 ↔ health impairment age 35	−0.16	−0.19	−0.13	0.01	0.000	−0.47

→ denotes a regression path; ↔ denotes a covariation/correlation

The text in bold shows the key parameters

Next, satisfaction with life and health, and health impairment at the age of 35 were included (M9). Specifically, somatic symptoms at the age of 45 were regressed on satisfaction with life and health and health impairment at the age of 35, and, in turn, regressed the three variables on the intercept and slope factors on adolescent somatic symptoms and negative emotional reactivity. This model is illustrated in Fig. 1. It shows a good fit to the data (Table 2). Both the intercept and slope factors of somatic symptoms and the intercept factor of negative emotional reactivity remained significantly associated with somatic symptoms at the age of 45, over and above satisfaction with life, satisfaction with health, and health impairment (Table 4). As can be seen in Table 4, the intercept and slope factors of the adolescent processes were differentially related to the three variables at the age of 35. For instance, those participants who reported higher levels in somatic symptoms and negative emotional reactivity in adolescence and increased in somatic symptoms reported lower satisfaction with their health in adulthood. In contrast, those participants with higher levels and increases in negative emotional reactivity across the adolescent years were those who reported lower satisfaction with life in adulthood. Health impairment though was only associated with the level of somatic symptoms in adolescence.

### Sensitivity Analyses

In a final step, the two models (M8, M9) were reran with gender (1 = female, 2 = male) as a time-invariant covariate (M10, M11). The model fit for both models are shown in Table 2. As expected, gender was negatively significantly associated with the levels and slopes of somatic symptoms and negative emotional reactivity in both models (Table 5). That is, adolescent girls reported more somatic symptoms and higher negative reactivity than boys early in adolescence. Moreover, the negative gender effects on the change parameters indicate that somatic symptoms and negative emotional reactivity showed differential development across the adolescent years with stronger increases in adolescent girls as compared to boys. Finally, the results indicate that female participants reported lower satisfaction with life at the age 35 than male participants (Table 5). However, no gender differences were found for satisfaction with health and health impairment at age 35, or somatic symptoms at the age 45. Apart from these gender effects, the results show that the main outcomes reported above are broadly similar when accounting for gender, with very few exceptions (Table 5). For instance, when including gender in Model M8, the slope of negative emotional reactivity in adolescence was no longer a significant predictor of somatic symptoms at age 45 (M10 in Table 5).

**Table 5:** Selected parameter estimates from the bivariate conditional LCM-SR for somatic symptoms and negative emotional reactivity in adolescence and adult health and wellbeing controlling for gender effects (Models M10 and M11)

Parameter	Estimate	95% CI LL	95% CI UL	*SE*	*p*	Std. Est.
*Model M10*						
Intercept of somatic symptoms → somatic symptoms age 45	0.32	0.18	0.45	0.07	0.000	0.21
Intercept of negative emotional reactivity → somatic symptoms age 45	0.39	0.16	0.61	0.12	0.001	0.14
Slope of somatic symptoms → somatic symptoms age 45	1.85	0.88	2.83	0.50	0.000	0.27
Slope of negative emotional reactivity → somatic symptoms age 45	1.45	−0.36	3.26	0.92	0.12	0.11
Gender → intercept of somatic symptoms	−0.21	−0.27	−0.15	0.03	0.000	−0.27
Gender → intercept of negative emotional reactivity	−0.05	−0.08	−0.02	0.02	0.002	−0.11
Gender → slope of somatic symptoms	−0.04	−0.06	−0.03	0.01	0.000	−0.26
Gender → slope of negative emotional reactivity	−0.03	−0.04	−0.02	0.01	0.000	−0.37
Gender → somatic symptoms age 45	−0.08	−0.17	0.02	0.05	0.10	−0.07
*Model M11*						
Intercept of somatic symptoms → satisfaction with life age 35	−0.27	−0.53	−0.003	0.13	0.04	−0.09
Intercept of somatic symptoms → satisfaction with health age 35	−0.27	−0.43	−0.11	0.08	0.001	−0.15
Intercept of somatic symptoms → health impairment age 35	0.13	0.02	0.23	0.05	0.02	0.11
**Intercept of somatic symptoms → somatic symptoms age 45**	0.26	0.13	0.38	0.07	0.000	0.17
Slope of somatic symptoms → satisfaction with life age 35	−0.49	−2.04	1.05	0.79	0.53	−0.04
Slope of somatic symptoms → satisfaction with health age 35	−1.09	−2.04	−0.14	0.48	0.02	−0.13
Slope of somatic symptoms → health impairment age 35	0.14	−0.46	0.73	0.30	0.66	0.03
**Slope of somatic symptoms → somatic symptoms age 45**	1.63	0.74	2.53	0.46	0.000	0.24
Intercept of negative emotional reactivity → satisfaction with life age 35	−1.12	−1.57	−0.68	0.23	0.000	−0.20
Intercept of negative emotional reactivity → satisfaction with health age 35	−0.38	−0.65	−0.11	0.14	0.006	−0.11
Intercept of negative emotional reactivity → health impairment age 35	0.16	−0.02	0.34	0.09	0.07	0.07
**Intercept of negative emotional reactivity → somatic symptoms age 45**	0.25	0.04	0.46	0.11	0.02	0.09
Slope of negative emotional reactivity → satisfaction with life age 35	−4.86	−8.36	−1.37	1.78	0.006	−0.19
Slope of negative emotional reactivity → satisfaction with health age 35	−1.07	−3.07	0.92	1.02	0.29	−0.07
Slope of negative emotional reactivity → health impairment age 35	0.07	−1.20	1.35	0.65	0.91	0.01
**Slope of negative emotional reactivity → somatic symptoms age 45**	1.02	−0.67	2.71	0.86	0.24	0.08
Satisfaction with life age 35 → somatic symptoms age 45	−0.06	−0.09	−0.03	0.02	0.000	−0.13
Satisfaction with health age 35 → somatic symptoms age 45	−0.12	−0.17	−0.07	0.03	0.000	−0.15
Health impairment age 35 → somatic symptoms age 45	0.18	0.11	0.26	0.04	0.000	0.15
Satisfaction with life age 35 ↔ satisfaction with health age 35	0.35	0.30	0.40	0.02	0.000	0.44
Satisfaction with life age 35 ↔ health impairment age 35	−0.16	−0.19	−0.13	0.01	0.000	−0.31
Satisfaction with health age 35 ↔ health impairment age 35	−0.15	−0.17	−0.13	0.01	0.000	−0.47
Gender → intercept of somatic symptoms	−0.21	−0.27	−0.15	0.03	0.000	−0.27
Gender → intercept of negative emotional reactivity	−0.05	−0.08	−0.02	0.02	0.002	−0.12
Gender → slope of somatic symptoms	−0.04	−0.06	−0.03	0.01	0.000	−0.28
Gender → slope of negative emotional reactivity	−0.03	−0.04	−0.02	0.01	0.000	−0.36
Gender → satisfaction with life age 35	−0.35	−0.52	−0.18	0.09	0.000	−0.15
Gender → satisfaction with health age 35	−0.08	−0.19	0.02	0.05	0.11	−0.06
Gender → health impairment age 35	−0.01	−0.07	0.06	0.03	0.83	−0.01
Gender → somatic symptoms age 45	−0.10	−0.19	0.02	0.05	0.11	−0.09

→ denotes a regression path; ↔ denotes a covariation/correlation

The text in bold shows the key parameters

## Discussion

Few studies have examined bidirectional within-person associations between negative emotional reactivity and somatic symptoms in adolescence and their predictive associations with health and wellbeing in later adulthood. To address this gap, this study employed data from a longitudinal study with annually assessments at the age of 12 to 16 years and further assessments at the age of 35 and 45. Four important findings emerged. First, negative emotional reactivity and somatic symptoms were positively associated at the between-person and within-person levels during adolescence. Adolescents who reported higher somatic symptoms also reported higher reactivity, and changes in somatic symptoms were associated with changes in reactivity within adolescents. Second, both levels and changes in somatic symptoms and negative emotional reactivity during adolescence were significantly associated with adult somatic symptoms over three decades later. That is, adolescents higher on somatic symptoms and reactivity and those who increased on somatic symptoms and reactivity over time tended to report more somatic symptoms in middle adulthood. Third, the prospective associations of the level factors of somatic symptoms and reactivity, as well as the change in somatic symptoms during adolescence, with somatic symptoms in middle adulthood largely held when accounting for satisfaction with life and health, and health impairment at age 35. Fourth, the results were robust when accounting for gender as time-invariant covariate.

### Negative Emotional Reactivity and Somatic Symptoms during Adolescence

Somatic symptoms such as headache, stomach pain, and nausea may increase from childhood to adolescence and are highly prevalent in adolescence, reflecting adolescence as a challenging developmental phase of life (Campo, 2012). Consistent with this view, an average small increase in somatic symptoms from age 12 to 16 was observed. More importantly, adolescents differ significantly in their level of somatic symptoms at age 12 and their change over the adolescent years, as both the intercept and the rate of change show significant variability. For instance, in line with previous research, adolescents girls reported more somatic symptoms than boys, and they also showed an increase in symptomatic concerns during adolescence (Steinhausen & Winkler Metzke, 2007; Swain et al., 2014). In addition to mean-level changes, the rank-order stability coefficients showed relatively high stability at the between-person level from year to year, while the within-person autoregressive parameters indicated rather low stability of somatic symptoms within adolescents from year to year. These findings suggest that somatic symptoms do not reflect perfectly stable concerns in adolescence, and thus it is critical to capture changes over time.

In contrast to somatic symptoms, these results indicated that, on average, negative emotional reactivity was relatively stable during adolescence. That said, adolescents differed meaningfully in how emotionally reactive they were at baseline and how they develop during adolescence. Again, gender played a role in predicting individual differences, as adolescent girls reported more negative emotional reactivity than boys and showed an increase in reactivity during adolescence, whereas the boys tended to decrease in reactivity. These gender effects mirror the gender differences in the development of neuroticism during adolescence (cf. Soto & Tackett, 2015). Research has shown that while girls and boys show similar degrees of anxiety and sadness throughout childhood using self-reports, substantial gender differences emerge by late adolescence and persists into adulthood (Soto, 2016; Van den Akker et al., 2014). However, there is also some evidence that parents and adolescents may disagree on gender differences in the development of neuroticism. While self-reports in a recent longitudinal study followed the pattern of earlier work, the opposite pattern was observed in parent reports with higher neuroticism degrees observed in boys (Slobodskaya & Kornienko, 2021). As such, future research may wish to combine data from multiple reporters, particularly during the adolescent years.

Consistent with previous work on the links between neuroticism and subjective and objective health (Rosmalen et al., 2007; Stephan et al., 2020), the current results suggest that negative emotional reactivity is positively associated with somatic symptoms both at the between-person and within-person levels during adolescence. Adolescents who reported higher somatic symptoms also reported higher negative emotional reactivity, and changes in somatic symptoms during adolescents were associated with changes in reactivity within adolescents. Overall, the present study replicates previous findings on the association between neuroticism and somatic symptoms but with a focus on one aspect of neuroticism, namely the tendency to react to unpleasant events and stressors with negative emotions. It should be noted that, in addition to reactivity, neuroticism primarily refers to the general tendency to experience negative emotions frequently and intensely (Friedman, 2019). Hence, future research is needed that simultaneously includes measures of negative emotional reactivity and neuroticism to examine the unique effects of the two constructs with respect to somatic concerns.

The results also suggest that negative emotional reactivity and somatic symptoms are related in terms of concurrent associations and correlated changes, but not related in terms of within-person cross-lagged effects. This indicates that within the same person, changes in somatic symptoms did not predict changes in negative emotional reactivity (and vice versa) at subsequent measurement occasions. The lack of within-person cross-lagged effects may imply, for instance, that increases in somatic symptoms are not impactful enough to shape changes in negative emotional reactivity or that increases in negative emotional reactivity does not necessarily lead to more somatic symptoms. Perhaps the annual time interval between the assessments was too long to capture cross-lagged effects. Given that associations are more dynamic at the within-person than between-person level, time intervals that are too short or too long based on the nature of the variables of interest can produce data that might be overly sensitive to measurement error or insensitive to variability and change (Hertzog & Nesselroade, 2003). It is possible that on days when somatic symptoms are more pronounced than average, this influences negative emotional reactivity at that moment or on that day. For example, a daily diary study across three weeks in an adult sample showed a cross-lagged effect of pain on negative affect the next day (Katana et al., 2020). Conversely, a stronger temporary expression of negative emotional reactivity than average could also increase the experience of somatic symptoms or lead to an inflated reporting of somatic symptoms. Future research may wish to investigate bidirectional and cross-lagged processes between somatic symptoms and negative emotional reactivity in adolescents using ambulatory assessments or daily diary studies.

Future research is also needed to understand the mechanisms between emotional reactivity and somatic symptoms. One possible candidate relates to the ability to regulate emotions (Gross, 2015). While negative emotional reactivity describes how quickly people tend to respond with negative emotions to stressors, unpleasant events and challenges, emotion regulation processes describe how quickly people are able to down-regulate the negative emotions. The ability to effectively regulate negative emotions reduces the extent to which people remain in negative affective states and may also dampen the experience of somatic symptoms. In contrast, difficulties with emotion regulation are likely to prevent people from disengaging from negative states, which can amplify the experience of somatic symptoms. There is increasing evidence from research in general and clinical populations that difficulties in emotion regulation are associated with more somatic symptoms (Schnabel et al., 2022).

### Long-Term Associations with Adult Health and Wellbeing

Somatic symptoms and negative emotional reactivity have been associated with adverse psychological and social consequences in adolescence, including absence from school, reduced academic achievement, and impaired work ability (Basch et al., 2015; Winding & Andersen, 2019). These consequences provide insights into the current findings, that they also predict adult health and wellbeing in early adulthood and middle adulthood. Adolescence and early adulthood are important years to lay foundations for health and health decisions that may determine health and wellbeing trajectories across the lifespan (Mroczek et al., 2020; Patton et al., 2016). Consistent with this view, the results indicate that individual differences in the levels of somatic symptoms and negative emotional reactivity are associated with satisfaction with life and health at age 35, as well as somatic symptoms at the age of 45. One explanation is that the issues incurred during adolescence, such as reduced progress at school and work, may set a course for frustration and difficulties catching up with peers in the years ahead. Another possibility is that adolescent risk-taking and healthcare decisions could yield future health issues in adulthood, as actions like substance use can hold long-term somatic consequences. Finally, adults who experience greater somatic symptoms in adolescence also were more likely to experience impaired health in adulthood. These findings regarding the long-term effects of high levels of somatic symptoms and negative emotional reactivity are consistent with research showing that somatic complaints can predict mental health problems and lower wellbeing later in life, including severe mental illness (Bohman et al., 2018; Shanahan et al., 2015) and delayed development of compassion in adulthood (Saarinen et al., 2020). As such, future research should consider how early health concerns chart a course for poorer wellbeing, and whether these associations depend on the development over time and endemic nature of the health concerns in adolescence.

The need for investigating consistency and change in somatic symptoms is evident given the current findings that changes during adolescence uniquely predict later adult outcomes. This study thus makes an important contribution by demonstrating that individual differences in change in somatic symptoms and negative emotional reactivity during adolescence were independently associated with long-term adult health and wellbeing decades later in life. Such work supports the suggestion that capturing how adolescents mature and develop during adolescence may be critical to understanding their later life outcomes (Hill et al., 2019). Moreover, this study provides further evidence for the role of individual changes in traits and competencies during adolescence as predictors of later health adult outcomes (Allemand et al., 2022; Steiger et al., 2014).

The present findings have practical implications. Given that an individual’s somatic symptoms and negative emotional reactivity may change during the adolescent years, and individual differences in change may affect adult health and wellbeing, it appears that early prevention and intervention programs are particularly promising to promote healthy youth development. Prevention of somatic symptoms in adolescence can include lifestyle modification and promotion of self-care, including stress management, practice physical activity, and participation in social activities (Duberg et al., 2013; van Sluiijs et al., 2007). Moreover, targeting negative emotional reactivity appears to be a potentially viable route to promote health and wellbeing in adulthood. Indeed, recent research has shown that neuroticism, which involves negative emotional reactivity, can be modified by clinical treatments (Roberts et al., 2017) and nonclinical interventions (Allemand & Flückiger, 2022; Stieger et al., 2021). Since somatic symptoms and negative emotional reactivity were interrelated in this study, one would expect that affecting one construct might also have an impact on the other construct. Applied research is needed to test this idea.

### Limitations and Future Directions

The current study has several important strengths including a large sample, and a unique longitudinal design ranging from adolescence to middle adulthood, and covering a time span of almost four decades. However, it is limited in ways that should promote future research. One limitation is that the current study relied solely on self-reports. Future research would benefit from including observer-reports and behavioral measures, although it is worth noting that self-report biases are less likely to explain associations spread several years apart. Moreover, the alpha reliability estimates of the measure of somatic symptoms were below 0.70 at ages 12 to 14 with the lowest alpha estimate of 0.66 at age 12. Furthermore, the measure of somatic symptoms only captured the frequency of somatic symptoms, not the severity of symptoms or how much life was affected by symptoms. It would be valuable to conduct future research with a measure that captures both the frequency and perceived severity of somatic symptoms. Moreover, given the timing of the original data collection, the common personality trait taxonomies of today were unavailable; as such, future research may wish to examine whether negative emotional reactivity holds unique associations with adult health and wellbeing outcomes compared to neuroticism. As is the case in all observational studies, it is not possible to rule out the possibility of unknown or unmeasured confounders. Likewise, somatic symptoms reported at middle adulthood may have different origins than those reported during adolescence, such as new-onset somatic diseases. As such, it would be valuable to examine predictors of somatic symptoms and negative emotional reactivity specifically for adolescence, early adulthood, and middle adulthood. Finally, the current study focused on participants from Germany. It is not immediately clear from a theoretical perspective why one would expect cultural or historical differences in the long-term associations evidenced. However, work is needed to understand the generalizability of findings beyond this relatively culturally homogenous sample that uses additional cohorts.

## Conclusion

Although previous studies have examined the relationship between neuroticism and health, particularly in adults, less is known about how negative emotional reactivity as an aspect of neuroticism is related to reports of somatic symptoms in adolescents. The current findings contribute to this underrepresented research in four important ways. First, this study has shown that adolescents with frequent somatic symptoms have higher negative emotional reactivity than adolescents with fewer symptoms. Second, adolescents differ from one another in their level and change of somatic symptoms and negative emotional reactivity during adolescence. Third, those individual differences in levels and changes were independently associated with adult health and wellbeing outcomes decades later. Fourth, although adolescent girls showed higher levels and greater increases in somatic symptoms and negative reactivity than boys, the results linking adolescent development to adult outcomes were robust. The current findings suggest that it would be useful to address somatic symptoms and/or negative emotional reactivity in prevention and intervention programs to promote positive adolescent development and ultimately adult health and wellbeing.

## Appendix

Table 6

**Table 6:** Items of the negative emotional reactivity scale in German (Fend, 1994; Fend & Prester, 1986) and English

Original in German	English translation
1. Ich rege mich manchmal über jede Kleinigkeit auf.	1. Sometimes I get upset about every little thing.
2. Gegen meine Launen komme ich manchmal kaum an.	2. Sometimes I can hardly fight against my moods.
3. Ich gehöre zu denen, die sich vor Wut manchmal nicht beherrschen können.	3. I am one of those who sometimes can’t control themselves because of anger.
4. Manchmal weiss ich gar nicht, was mit mir eigentlich los ist.	4. Sometimes I don’t know what’s going on with me.
5. Es gibt Tage, an denen mir jeder auf die Nerven geht.	5. There are days when everyone gets on my nerves.
6. Manchmal bin ich ohne wichtigen Grund sehr betrübt.	6. Sometimes I feel very sad for no important reason.
7. Wenn mich etwas ärgert, kann ich mich manchmal völlig vergessen.	7. When something annoys me, I can sometimes forget myself completely.
8. Manchmal ist mir alles völlig egal.	8. Sometimes I don’t care about anything at all.

The English translation reflects a translation with an online translator

## Footnotes

1 Previous articles have used data from the LifE-Study to examine the long-term effects of the development of self-control (Allemand et al., 2019; Allemand et al., 2023), self-esteem (Gruenenfelder-Steiger et al., 2016; Steiger et al., 2014, 2015), empathy (Allemand et al., 2015), and perceptions of the future (Allemand et al., 2022) during adolescence. The constructs investigated in the current study were not investigated in these previous studies.
